# Assessment of galectins -1, -3, -4, -8, and -9 expression in ovarian carcinoma patients with clinical implications

**DOI:** 10.1186/s12957-022-02738-4

**Published:** 2022-09-01

**Authors:** Radwa Mansour Mohamed, Athar Emam, Mahmoud M. Abdelfattah, Abdel-Mageed Ismail Abdel-Mageed, Mohamed A. Abdelhafeez, Reham Helwa

**Affiliations:** 1 Department of Obstetrics and Gynaecology, Faculty of Medicine, Ain Shams University, Cairo, Egypt; 2 Molecular Cancer Biology group, Zoology Department, Faculty of Science, Ain Shams University, Cairo, Egypt

**Keywords:** Ovarian carcinoma, Galectins, mRNA, qRT-PCR, Biomarkers, Lymph node metastasis

## Abstract

**Background and aim:**

Galectins have been recently tackled by many researchers in the field of cancer due to their role in tumorigenesis, disease progression, and metastasis. Thus, they are currently involved in biomarkers research on several types of cancer. In ovarian cancers, few studies were carried out to evaluate galectins expression profiling. Hence, our present study was executed to evaluate the mRNA expression of galectins -1, -3, -4, -8, and -9 in epithelial ovarian cancers.

**Methods:**

Fifty-six tumor samples of ovarian carcinomas were analyzed for mRNA expression using qRT-PCR, and fold-changes were calculated in comparison to tissue samples of 26 women with normal ovaries.

**Results:**

The results of the present paper emphasize the importance of galectins as predictors for targeted therapy. *LGALS1*, *LGALS3*, *LGALS4*, *LGALS8*, and *LGALS9* were found to be mostly overexpressed in ovarian carcinoma patients with the following percentage: 78.6%, 92.9%, 66.1%, 87.5%, and 85.7% respectively. Moreover, galectins -3 and -9 were found to be significantly elevated with lymph node metastasis (*p* = 0.044 and *p* = 0.011). Also, upregulation of galectin-1 and -9 were statistically significant in stages IIB, IIC, and IIIB (*p* = 0.002) in FIGO staging. CA19.9 is positively correlated to galectin-4 expression (*p* = 0.039).

**Conclusion:**

Our findings strengthen the role of galectins in carcinogenesis, disease progression, and lymphnode metastasis in ovarian carcinomas. And since these galectins are mostly overexpressed, they could be promising markers for targeted therapy to reduce disease progression and metastasis process.

**Supplementary Information:**

The online version contains supplementary material available at 10.1186/s12957-022-02738-4.

## Introduction

According to global cancer observatory, ovarian cancer in Egypt is one of the top 5 most frequent cancers excluding non-melanoma skin cancer, ranked by number of cases in women [1]. Globally, ovarian cancer is the third most common gynecological cancer after cancer cervix and uterine cancer, but the second in mortality rate [2]. Nevertheless, cervical cancer is considered completely preventable due to the possibility of screening and early treatment; ovarian cancer lacks proper screening strategy as well as sharing non-specific symptoms with other benign gastro-intestinal diseases, which makes it difficult to diagnose and cure. Therefore the majority of women present with late disease stages, and survival rates have not improved compared to other gynecological cancers [3].

Galectins are family of animal lectins which are involved in several cancer aspects including carcinogenesis, tumor growth, cell migration, angiogenesis, invasion, and metastasis. They are likely used as prognostic markers [4–8]. It was found that galectins are interacting molecules with red blood cells via blood group antigens which could act as a potential pathway in metastasis [9]. Significant elevation of galectins expression was previously reported in several types of cancers including gynecological tumors [6, 10–15]. The overexpression of galectins and their role in tumorigenesis/cancer progression render them as potential therapeutic targets [16]. All galectins have at least one carbohydrate recognition domain (CRD) which binds specifically to sugar (β-galactoside) [17]. Accordingly, several researches target the CRD as it is the functional domain of all galectins. Currently, there are several classes of directed therapeutics to target galectins including small non-carbohydrate, inhibitors (e.g., competitive carbohydrate), and antibodies [16, 18, 19].

In the present study, we aim at investigating the mRNA expression of five galectins (*LGALS1*, *LGALS3*, *LGALS4*, *LGALS8*, and *LGALS9*) in epithelial ovarian carcinoma patients to assess their roles as biomarkers for disease progression and potential therapeutic targets.

## Materials and methods

### Patients’ samples and clinicopathological data

A fresh ovarian tissue sample of about 2 × 2 cm was collected from 56 diagnosed adult female patients with epithelial ovarian cancer; confirmed by final pathological examination with median age of 48.5 years. The patients were recruited from Ain-Shams University Maternity hospital after signing an approval consent to share in the study, and the study procedures were approved by the ethical committee. Twenty-six benign ovarian tissue samples (Supplementary Table 1) were also taken for normalization. Samples were collected immediately after excision of the tissues on the day of the patients’ planned surgery either for treatment of ovarian cancer or for a benign indication. The tissues were preserved in RNA Later (Qiagen, Germany) and stored at – 80 °C until the RNA purification step takes place. As regards the patients’ demographic data, 41 patients were premenopausal and 15 were postmenopausal. All the patients underwent total abdominal hysterectomy and bilateral salpingo-oophorectomy with infra-colic omentectomy, pelvic lymph node resection, and excision of any visible disease. All the patients had residual disease of < 1 cm; indicating complete cytoreduction. Final histopathological examination revealed 27 epithelial cancer of serous type; 23 mucinous adenocarcinoma and 6 clear cell carcinoma of the ovary. Nineteen samples showed positive lymph nodes. CA125 and CEA were elevated in all patients, with only 37 positive CA19.9.

### RNA isolation, cDNA synthesis, and qRT-PCR

Total RNA was purified from the freshly collected tumors using TRIZOL reagent (Bioflux, China) following the manufacturer’s protocol. cDNAs were synthesized using MMLV reverse transcriptase (Genedirex). PCR was performed for five Galectins (1, 3, 4, 8, and 9) and GAPDH genes using specific primers. The primers’ sequences were the same as previously published article [15]. Stratagene Mx3000P was used to execute the cycling conditions which was as the following: (1) initial denaturation at 95 °C for 10 min, (2) 40 cycles of 95 °C for 15 s, annealing at 60 °C for 30 s, and extension for 60 s, and finally dissociation was also performed to check melting curves. The experiments were repeated 3 times for consistency and reproducibility.

Several previous studies have been using GAPDH as a reference gene. According to Cadenas, J et al. (2022), GAPDH was found to be the most stable gene for this type of tissue [20]. Also, according to Nikishin et al., they found that levels of GAPDH, ACTB, and HSP90 proteins are similar in native and vitrified/thawed ovarian samples [21]. The CT (cycle threshold) data were extracted on excel sheets and normalized to *GAPDH*. The fold change analysis was carried out using 2^−ΔΔCT^ formula.

### Statistical analysis

Data was analyzed using IBM SPSS statistical software version 24 (SPSS Inc., Chicago, IL, USA). Kolmogorov-Smirnov and Shapiro-Wilk normality tests were performed to examine whether a variable was normally distributed. The values of the normality tests were below 0.05. ANOVA was performed for a continuous independent variable. A Mann-Whitney and Kruskal-Wallis tests were used to compare galectins expression and clinical subgroups of patients. A value of *p* < 0.05 was considered statistically significant.

Receiver-operating characteristic (ROC) curve analysis is used to examine the predictive value of galectins expression. The area under the ROC curve (AUC) is interpreted as follows: AUC < 0.6 = fail, 0.6 to 0.69 = poor, 0.7 to 0.79 = fair, 0.8 to 0.89 = good, ≥ 0.9 = excellent. We applied the Bonferroni correction to adjust the type I error for multiple comparisons. *p* values < 0.006 are considered statistically significant for ROC analysis.

## Results

### Galectins -1, -3, -4, -8, and -9 expression in ovarian carcinomas

**Fig. 1:**
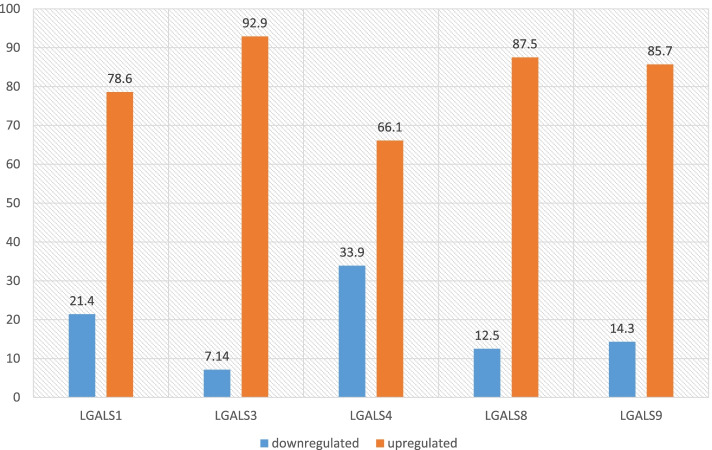
Up- and downregulation of 5 galectins in ovarian carcinomas

Fifty-six ovarian carcinomas were analyzed using qRT-PCR to evaluate the mRNA expression of five galectins. The obtained CT values were normalized to GAPDH. Then, the fold change was calculated by comparing samples to 26 samples of normal ovaries. Accordingly, the five galectins -1, -3, -4, -8, and -9 were mostly upregulated in OC patients as the following: 78.6%, 92.9%, 66.1%, 87.5%, and 85.7% respectively as shown in Fig. 1.

### Galectins expression versus clinicopathological data

The fold changes of gene dysregulation were analyzed for every item in the clinicopathological data. The results are shown in Tables 1 and 2. According to our results, galectins expression was significantly associated with some clinical aspects.

**Table 1:** Clinicopathological data of the studied cohort

Parameter	Number (***n***)			Percent (%)		
**FIGO 2014 Classification**						
Stage IA	0			0%		
IB	0			0%		
IC	20			35.7%		
Stage IIA	0			0%		
IIB	11			19.6%		
IIC	6			12.5%		
Stage IIIA	0			0%		
IIIB	8			14.2%		
IIIC	7			12.5%		
Stage IV	4			7.14%		
**Age (years)**						
< 50	29			52%		
> 50	27			48%		
**Parity**						
Nulliparous	2			3.6%		
Multipara	54			96.4%		
**Menopausal status**						
Post-menopausal	15			26.8%		
Premenopausal	41			73.2%		
**Organ metastasis**						
Present	4			7.14%		
Absent	52			92.9%		
**Lymph node metastasis**						
Present	19			33.9%		
Absent	37			66.1%		
**Cytology types**	Number			Percent		
Mucinous	23			41.1%		
Serous	27			48.2%		
Endometroid	0			0%		
Clear cell carcinoma	6			10.7%		
Brenner	0			0%		
Small cell	0			0%		
**Tumor markers**	High		Normal		Not available	
	*N*	%	*N*	%	*N*	%
CA125	50	89%	6	11%	0	0
CEA	46	82%	10	18%	0	0
CA19.9	13	23%	24	43%	19	34%

**Table 2:** Statistical analysis of five galectins expression versus clinicopathological data

Clinical data	p value				
	***LGALS1***	***LGALS3***	***LGALS4***	***LGALS8***	***LGALS9***
**Age**	0.587	0.439	0.890	0.360	0.412
**parity**	0.332	0.005	0.005	0.519	0.469
**menarche**	0.747	0.543	0.074	0.625	0.367
**BMI**	0.299	0.325	0.363	0.146	0.161
**Menopause**	0.948	0.281	1	0.662	0.261
**Medical comorbidities**	0.005	0.206	0.008	0.015	0.071
**Surgical comorbidities**	0.332	0.005	0.005	0.519	0.469
**ca125**	0.126	0.39	0.115	0.425	0.490
**CEA**	0.846	0.438	0.484	0.677	0.623
**AFP**	0.377	0.591	0.374	0.394	0.378
**ca19.9**	0.757	0.431	0.039	0.481	0.672
**Organ metastasis**	0.414	0.818	0.206	0.591	0.396
**lymph node metastasis**	0.228	0.044	0.088	0.178	0.013
**FIGO STAGING**	0.002	0.575	0.426	0.156	0.002
**Cytology**	0.060	0.777	0.416	0.922	0.912

**Fig. 2:**
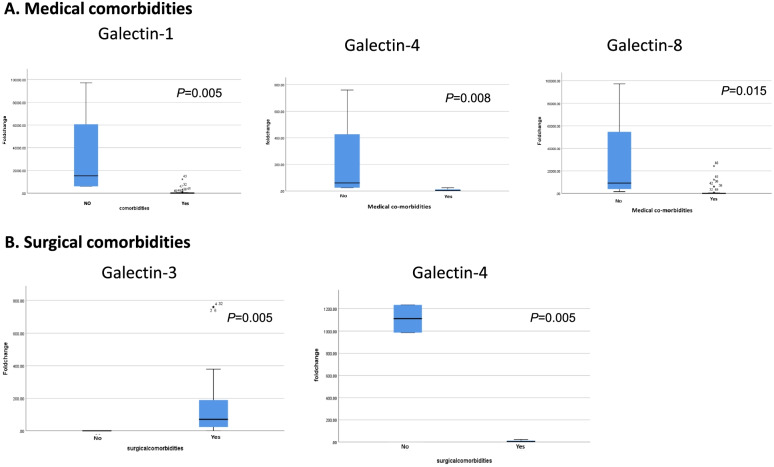
Galectins expression with medical and surgical comorbidities

The absence of medical comorbidities (as diabetes, hypertension, heart diseases or other diseases) was significantly associated with upregulation of galectins -1, -4, and -8 with *p* value = 0.005, 0.008, and 0.015 respectively (Fig. 2A). Galectin-4 was also found to be more abundant in patients without surgical comorbidities (*p* = 0.005). In contrast, high expression of galectins-3 was associated with surgical comorbidities (*p* = 0.005) as shown in Fig. 2B.

**Fig. 3:**
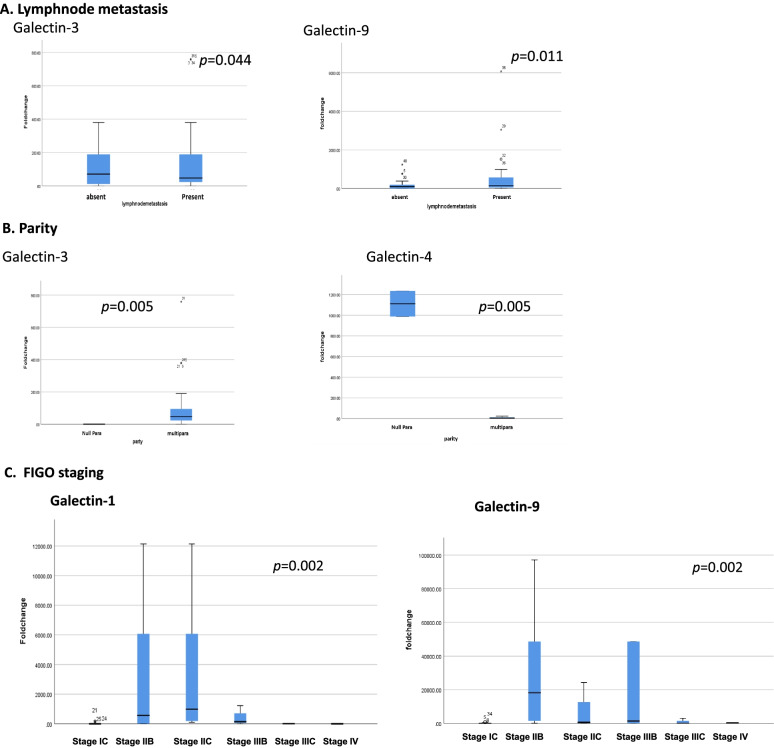
Galectins expression is significant with different clinical data. **A** lymph node metastasis, **B** parity, and **C** FIGO staging

Furthermore, an association between lymph node metastasis and upregulation of galectins -3 (*p* = 0.044) and -9 (*p* = 0.011) was detected. There was a tendency of association between galectins-4 and lymph node metastasis (*p* = 0.088) as well, but it didn’t reach the statistical significance (Fig. 3).

Parity among this data was proved to influence galectins expression. In the present cohort, surge of gallectin-3 expression was found in multiparaous (*p* = 0.005), whereas down-expression of galectins-4 was found in nulliparous (*p* = 0.005) as shown in Fig. 3B.

Kruskal-Wallis analysis of the five galectins and FIGO staging revealed a significant association of galectins-1 and -9 (*p* = 0.002) with higher FIGO stages. Figure 3C shows upregulation of galectins-9 to be significant in stages IIB, IIC, and IIIB.

**Fig. 4:**
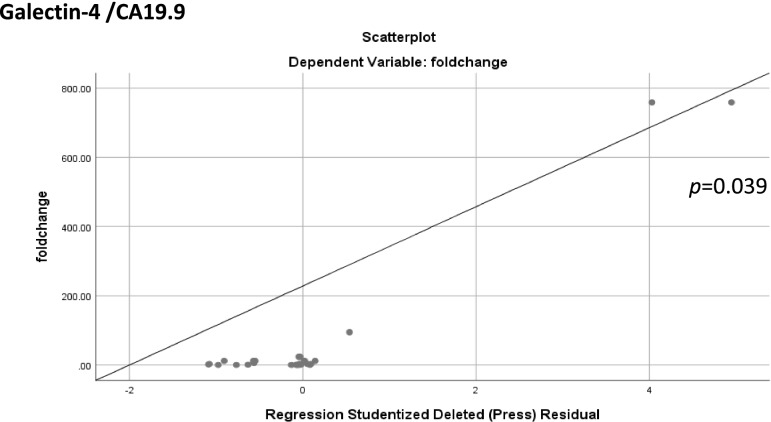
Linear regression analysis shows a positive correlation between galectin-4 and CA19.9

Linear regression analysis was also carried out to detect any correlation between galectins expression and tumor markers (CA125, CA19.9, and CEA). A significant positive correlation was found between galectin-4 and CA19.9 (*p* = 0.039) as shown in Fig. 4.

One of the results perceived showed a tendency of mRNA upregulation of galetin-1 in serous and clear cells subtypes (*p* = 0.060) as shown in supplementary Fig. 1.

### Galectin-9 could be used as predictive markers for LN metastasis

**Fig. 5:**
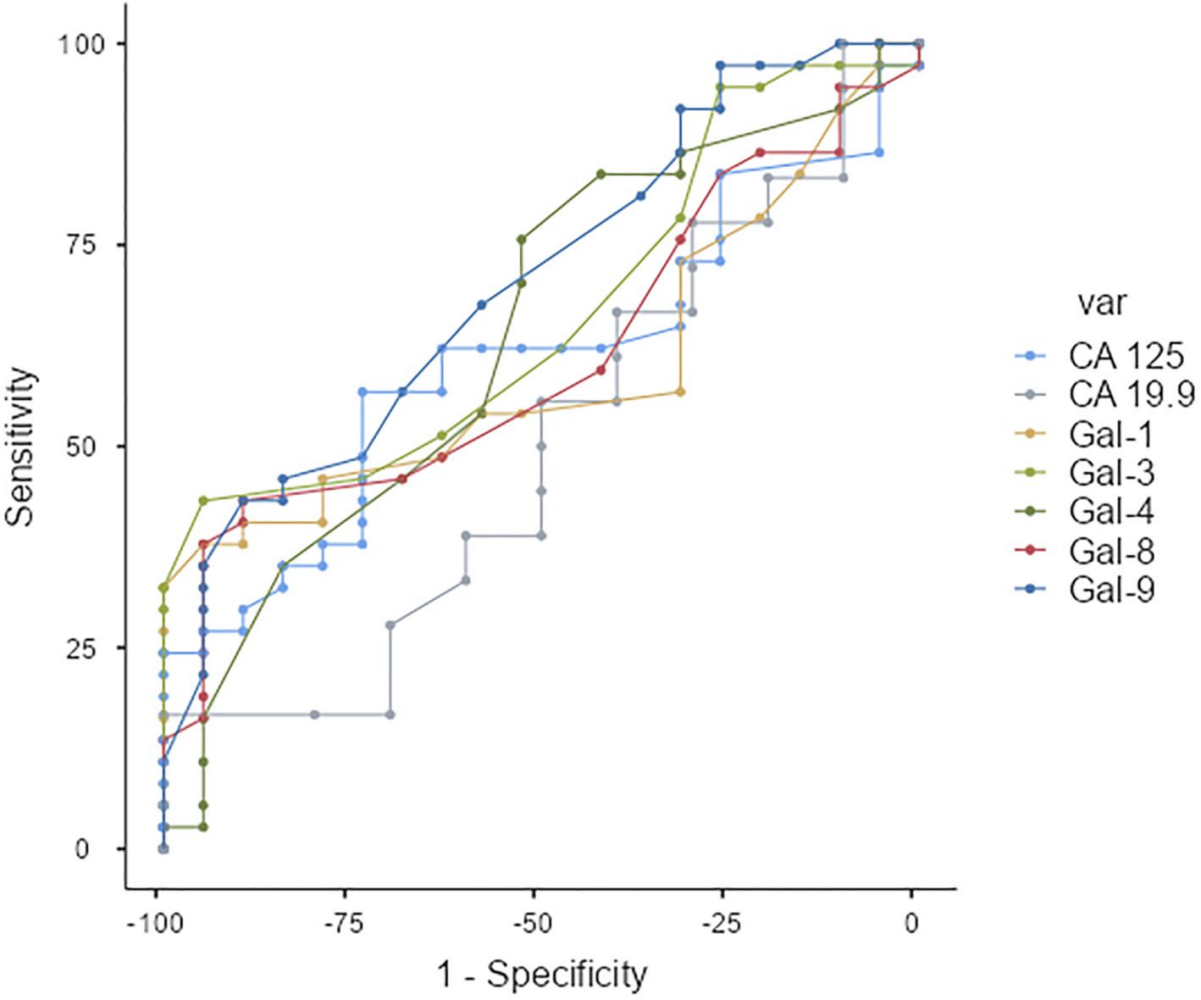
Response operating characteristic (ROC) curves for prediction of LN metastasis using galectins, CA 125 or CA 19.9

ROC analysis was performed to examine the diagnostic/predictive value of biomarkers as shown in Fig. 5 and Table 3. Galectin-9 was correlated with lymph node metastasis with 89% specificity and fair area under the curve (AUC) = 0.7 (*p* value (AUC = 0.5) = 0.005). However, the sensitivity was 43%. This result could be confirmed in the future by using bigger cohort.

**Table 3:** Response Operating Characteristic (ROC) curve analysis for prediction of LN metastasis using galectins, CA 125 or CA 19.9

	Marker						
ROC parameter	Gal-1	Gal-3	Gal-4	Gal-8	Gal-9	CA 125	CA 19.9
AUC	0.599	0.665	0.639	0.610	0.703	0.608	0.503
SE	0.076	0.073	0.080	0.076	0.072	0.076	0.120
95% CI	0.459 to 0.728	0.526 to 0.786	0.500 to 0.763	0.471 to 0.738	0.566 to 0.818	0.469 to 0.736	0.309 to 0.696
*z* statistic	1.300	2.257	1.747	1.448	2.826	1.418	0.023
*P* value (AUC = 0.5)	0.194	0.024	0.081	0.148	0.005	0.156	0.982
Youden index J	0.33	0.38	0.28	0.33	0.33	0.304	0.167
Associated criterion	> 1234.7	> 379.3	> 0.7	> 189.7	> 1234.7	> 134.1	> 150
Sensitivity (%)	38	43	76	43	43	57	17
Specificity (%)	95	95	53	89	89	74	100

*AUC* area under curve, *ROC* response operating characteristic, *SE* standard error

All *p* values are unadjusted as calculated with the DeLong method. *P* < 0.006 is considered statistically significant (Boneferroni method)

## Discussion

Galectins are among the molecules of interest in cancer biology. They are proteins which interact with sugar via their CRD. Thus, they are involved in several signaling pathways which start and/or promote cancer [5, 8]. Since these molecules are involved and over-expressed in many cancers, they are now investigated as potential therapeutic targets [16, 19]. In ovarian carcinomas, most of the previous studies focused on galectins -1, -3, -7, and -9, while ignoring the remaining 15 members of the family [6, 22]. In the present study, the mRNA expression of five galectins (*LGALS1*, *LGALS3*, *LGALS4*, *LGALS8*, and *LGALS9*) is explored in the tumor samples of 56 ovarian carcinoma patients, and the expression fold change was calculated in comparison to 26 adult females with normal ovaries. Of note, although it is universally well known that serous carcinomas of the ovaries are the most common histological subtype [23]; in our cohort, the number of serous and mucinous carcinomas were nearly the same. Gastrointestinal origin for mucinous carcinomas has been excluded by inevident clinical symptoms, upper and lower gastro-intestinal endoscopies, examination during surgery, and immuno-staining if needed.

After the analysis of fold changes, upregulation in the mRNA level of *LGALS1*, *LGALS3*, *LGALS4*, *LGALS8*, and *LGALS9* was detected in cancer patients compared to normal ovarian tissue. The most frequent upregulated galectin in OC was galectins-3; its overexpression in 92.9% of our cohort was significantly noted. In fact, this predominant overexpression of galectin-3 in OC patients increases the need for targeted therapy, especially that this overexpression was significantly associated with lymph node metastasis. This result is consistent with the function of galectin-3 and its increasing level in several types of cancers as published previously in several studies [6, 24–27]. These results are contradictory with what was found in two different cohorts of AML patients in Egypt, where galectin-3 was downregulated in AML patients [15, 28]. Both findings reflect the importance of retrospective studies to investigate gene expression profile in different cancers to receive certain treatment for precision/targeted therapy.

Galectin-8 was repeatedly studied in different types of cancer [29–31], but to the best of our knowledge, galectin-8 expression was not investigated in ovarian carcinomas patients. In our results, *LGALS8* mRNA has been found to be upregulated in 87.5% of the present cohort. This expression pattern indicate another potential molecular target for epithelial ovarian cancers.

Galectin-9 has been considered in a recent meta-analysis study as prognostic marker indicating poor overall survival rate in epithelial ovarian cancers [6]. In our cohort, high expression of LGALS9 was found in 85.7% of the patients. This upregulation was also associated with lymph node invasion reflecting a role of galectin-9 in disease progression. Using ROC analysis was found to be fair biomarker for lymph node metastasis. Additionally, LGALS9 upregulation was significantly presented in stages IIB, IIC, and IIIB. This result matches the result of an earlier study where galectin-9 overexpression was found to occur more often in low tumor stage and lower grading [7]. On the other hand, Schulz et al. found that overexpression of galectin-9 is associated with the best outcomes, while moderate expression was found to be correlated with reduced survival [32].

In a previous study on ovarian carcinomas, high galectin-1 was significantly observed in serous, clear cells and endometrioid types, while low galectin-1 was detected in mucinous type [7]. This data is in line with our results which show that there is a tendency of having high mRNA expression of galectin-1 in serous and clear cell carcinomas (*p* = 0.06). The insignificant *p* value could be attributed to the cohort size.

To the best of our knowledge, galectin-4 was not studied in ovarian carcinomas, but was reported in other types of cancers [15, 28, 33, 34]. Only one study examined galectin 4 in differentiation between primary ovarian mucinous carcinoma from gastrointestinal cancers that commonly metastasize to the ovaries [35]. In the present cohort, mRNA expression of *LGALS4* was upregulated in 66.1% of the patients with a positive correlation to CA19.9 (*p* = 0.039). This finding indicates a potential role of galectin-4 in ovarian carcinomas. Hence, it could be used as a biomarker for diagnosis/prognosis, as well as a therapeutic target.

## Conclusion

To sum up, galectins -1, -3, -4, -8, and -9 are mostly upregulated in ovarian carcinomas (78.6%, 92.9%, 66.1%, 87.5%, and 85.7% respectively), and galectins -3 and -9 are significantly high in lymph node metastasis (*p* = 0.044 and *p* = 0.011). Galectin-1 and -9 are overexpressed in stages IIB, IIC, and IIIB in FIGO staging (*p* = 0.002). Similarly, galectin-4 expression is positively correlated to CA19.9 (*p* = 0.039). Hence, these five galectins play a role in ovarian cancers that can act as a promising marker for targeted therapy, and/or that can help in the diagnosis/prognosis of the disease.

## Supplementary Information

**Additional file 1: Supplementary Figure 1.** Galetin-1 expression in different cytology status.

**Additional file 2: Supplementary Table 1.** Clinical parameters of control individuals (*N*=26).

## Data Availability

The datasets used and/or analyzed during the current study are available from the corresponding author on reasonable request.

## Abbreviations

cDNA: Complementary DNA
CRD: Carbohydrate recognition domain
mRNA: Messenger ribonucleic acid
OC: Ovarian cancer
qRT-PCR: Quantitative reverse transcription- polymerase chain reaction
ROC: Response Operating Characteristic
LN: Lymph node
AUC: Area under the curve
