# Supplementary figures and images for: Assessment of galectins -1, -3, -4, -8, and -9 expression in ovarian carcinoma patients with clinical implications
Source: World J Surg Oncol. 2022 Sep 1;20:276. doi: 10.1186/s12957-022-02738-4 (PMC9434928; doi:10.1186/s12957-022-02738-4)

## Slide 1
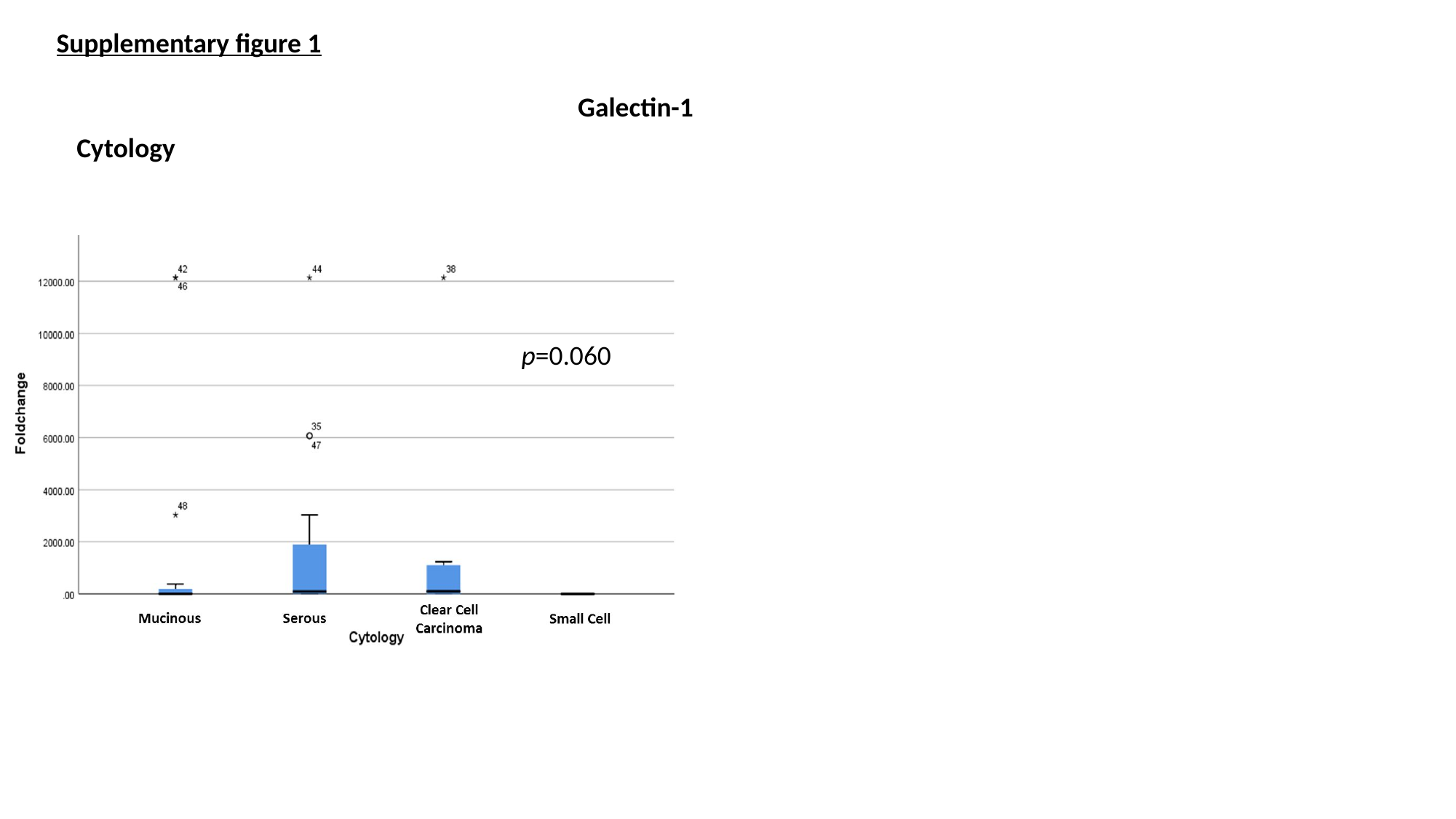

Supplementary figure 1
Galectin-1
Cytology
p=0.060

Supplement: Supplementary file 1 — Additional file 1: Supplementary Figure 1. Galetin-1 expression in different cytology status. [file 12957_2022_2738_MOESM1_ESM.pptx]
