# Supplementary material for: Assessment of galectins -1, -3, -4, -8, and -9 expression in ovarian carcinoma patients with clinical implications
Source: World J Surg Oncol. 2022 Sep 1;20:276. doi: 10.1186/s12957-022-02738-4 (PMC9434928; doi:10.1186/s12957-022-02738-4)
Supplement: Supplementary file 2 — Additional file 2: Supplementary Table 1. Clinical parameters of control individuals (N=26). [file 12957_2022_2738_MOESM2_ESM.docx]

Supplement Table1: clinical parameters of control individuals (N=26)

| **Age (years)** | | |
| --- | --- | --- |
| <50  >50 | 12  14 | 46%  54% |
| **Menopausal status** | | |
| Post-menopausal  Premenopausal | 8  18 | 31%  69% |
| **Parity** | | |
| Nulliparous  Multipara | 2  24 | 8%  92% |
| **Medical co morbidities** | | |
| Yes  No | 16  10 | 61.5%  38.5% |
| **Surgical co morbidities** | | |
| Yes  No | 17  9 | 65%  35% |
